# Case series: effects of platelet-rich plasma (PRP) on the recovery of bilateral muscle injuries after removal of semitendinosus and gracilis grafts in bilateral anterior cruciate ligament (ACL) reconstruction

**DOI:** 10.1093/jscr/rjae641

**Published:** 2025-02-10

**Authors:** Alberto De Castro Pochini, Anna Carolina Bueno, Roque Antonio Cury Mattos, Benno Ejnisman, Moises Cohen

**Affiliations:** Department of Orthopedics and Traumatology, UNIFESP, RUA BOTUCATU, 740-1o. ANDARVILA CLEMENTINO – SP, CEP 04023-900, Sao Paulo, Brazil; Sports Medicine and Physical Activity Discipline, EPM/UNIFESP, R. Estado de Israel, 713 - Vila Clementino, São Paulo - SP, 04022-002, Brazil; Knee Group, SBCJ (Hospital Novo Atibaia), R. Pedro Cunha, 145 - Vila Santista, Atibaia - SP, 12941-020, Sao Paulo, Brazil; Knee Surgery, Hospital Novo Atibaia, R. Pedro Cunha, 145 - Vila Santista, Atibaia - SP, 12941-020, Brazil; Center for Sports Traumatology - CETE, Federal University of São Paulo - UNIFESP, R. Estado de Israel, 713 - Vila Clementino, São Paulo - SP, 04022-002, Brazil; Medical Residency in Orthopedics and Traumatology, Santa Casa de Misericórdia de São Paulo, R. Dr. Cesário Mota Júnior, 112 - Vila Buarque, São Paulo - SP, 01221-010, Brazil; Orthopedic and Traumatology, Hospital São Francisco de Assis, R. 9-A, 110 - St. Aeroporto, Goiânia - GO, 74075-250, Brazil; Knee Surgery, Orthopedics, and Traumatology, Hospital Novo Atibaia, R. Pedro Cunha, 145 - Vila Santista, Atibaia - SP, 12941-020, Brazil; Sports Traumatology Section of the Department of Orthopedics and Traumatology, UNIFESP/EPM, R. Estado de Israel, 713 - Vila Clementino, São Paulo - SP, 04022-002, Brazil; Sports Medicine and Physical Activity Discipline of the Department of Orthopedics and Traumatology, UNIFESP/EPM, R. Estado de Israel, 713 - Vila Clementino, São Paulo - SP, 04022-002, Brazil; Department of Orthopedics and Traumatology, Paulista School of Medicine, Translational Surgery Postgraduate Program at UNIFESP (Medicine III, Capes grade 6), Postgraduate Program in Health Sciences at Hospital Israelita Albert Einstein, Rua Pedro de Toledo, 650 - Vila Clementino, São Paulo - SP, 04039-002, Brazil

**Keywords:** anterior cruciate ligament, platelet-rich plasma, muscle injury

## Abstract

To assess the healing of acute bilateral muscle injury in cases of bilateral anterior cruciate ligament reconstruction using platelet-rich plasma (PRP) after tendon graft removal. The study included 12 cases of bilateral anterior cruciate ligament reconstruction using semitendinosus and gracilis (STG) grafts. In the right knee, the STG graft was removed, and saline solution was applied; in the left knee, the graft was removed, and PRP was applied. Patients were evaluated using the visual analog scale, pre and postoperative isokinetic tests (5 months), and muscle area analysis. A slight difference in results was observed at 15 and 30 days on the PRP-treated side, but there was no variation in circumference and muscle strength. Due to the small sample size, the study will be continued to increase the number of cases, aiming for the publication of results.

## Introduction

Musculotendinous injuries are the most common injuries resulting from competitive physical activity, ranging from contusions due to direct trauma with a natural progression toward healing, to severe musculotendinous ruptures that, even after specific surgical or non-surgical treatment, may lead to significant sequelae and early discontinuation of athletic activity. Overloading physical activity represents the primary factor for injury in competitive athletes, whether due to insufficient time for injury recovery or inadequate treatment of previous injuries.

Morgan *et al*. [1], in a study with 237 soccer athletes during the 1996 season in the USA, found that the most frequently injured muscle group was the hip adductors (53%), followed by the hamstrings (42%) and quadriceps (5%). The most common injuries in the hip region in sports are muscle injuries. They occur more frequently in bi-articular muscles during eccentric movements and can happen at the musculotendinous junction or in the muscle belly. Adductor muscle injuries of the thigh frequently occur in soccer, hockey, and American football [2].

In a prospective study conducted from 1992 to 1999 in Australian football, which included a total of 2255 games and 672 hamstring muscle injuries, 163 quadriceps injuries, and 140 injuries to the calf muscles, several risk factors for muscle injuries were identified. These risk factors included previous muscle injuries, age (except for quadriceps injuries), and quadriceps injuries were more frequent in the dominant lower limb (kicking leg) [3].

A hamstring muscle injury is the most common injury among sprinting athletes, often resulting in prolonged absence from the sport and an increased risk of re-injury [4–7].

A muscle injury occurs when the tension generated exceeds the tensile capacity of the muscle’s weakest element. These injuries most frequently occur at the myotendinous junction or within the muscle belly. Muscle injuries can be caused by excessive internal force production, excessive external stretching, or both. Several factors contribute to a higher likelihood of muscle injury: inadequate stretching, insufficient muscle strength or endurance, dyskinetic muscle contraction, insufficient warm-up, or inadequate rehabilitation of a previous injury [8–10].

Hassleman *et al*. [11] demonstrated that there is an injury threshold caused by the active stretching of rabbit muscles. Initially, the injury occurs in the muscle fibers, followed by injury to the connective tissue, but only after significant muscle damage. In this study, the injury occurred both at the musculotendinous junction and within the muscle belly.

During intense physical activity, eccentric contraction is responsible for the majority of muscle injuries. The injury occurs when a strong muscle contraction is combined with muscle stretching (eccentric). The muscles at the highest risk for injury are the biarticular muscles (those that cross two joints).

PRP represents a volume of autologous plasma with a platelet concentration above the normal value. Normally, platelet counts range between 150 000 platelets/microliter and 350 000 platelets/microliter, with an average of 200 000 platelets/microliter. For the purpose of stimulating tissue healing, PRP is expected to have a concentration of 1000 000 platelets/microliter in 5 ml of plasma [2, 3].

The mechanism behind PRP in regenerative medicine has been well investigated and includes the identification and concentration of growth factors and exosomes released. The benefits of PRP have been highly recommended and widely used in orthopedics and sports medicine, including the repair of injured skeletal muscle [12].

Certain growth factors are secreted by platelets, including PDGF (Platelet-Derived Growth Factor), PGAF (Platelet-Derived Angiogenic Factor), TGF-α (Transforming Growth Factor Alpha), IGF-α (Insulin-like Growth Factor), VEGF (Vascular Endothelial Growth Factor), and EGF (Epidermal Growth Factor).

At the 5th Annual Hilton Head Workshop on Engineering Tissues in February 2001, it was suggested that TGF-α could be involved in fibrosis formation in muscle. Laboratory inhibition of TGF-α using suramin showed improvement in muscle structure after injury [13]. Additionally, clinical studies [11] have demonstrated that the use of PRP in muscle injuries reduced the average healing time from 22 days (in controls) to 16 days. Furthermore, meta-analysis studies have concluded that PRP improves pain in musculoskeletal injuries [14].

PRP is a potential treatment for some musculoskeletal diseases; however, the evidence of its efficacy has been highly variable, depending on the specific indication. Currently, due to restrictions on clinical use, PRP has been authorized only in scientific studies with approved ethics committees. Additional high-quality clinical trials with longer follow-up are crucial to shape our perspective on this treatment option [15].

Thus, this study aims to analyze the healing of acute muscle injuries associated with the use of platelet-rich plasma (PRP). It will involve patient follow-up and subsequently increase the number of cases in the study to provide more reliable research results.

## Case series

**Figure 1:**
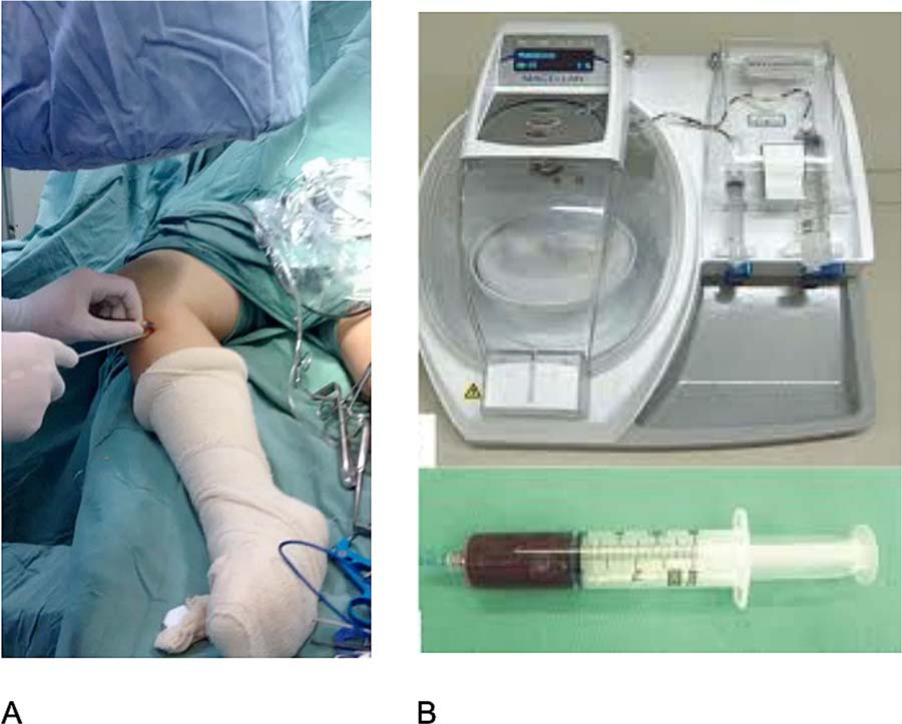
(A) STG removal under image intensifier; (B) PRP device.

This is a series of cases in which 12 patients with bilateral anterior cruciate ligament (ACL) injury were evaluated. The study was approved by the Brazilian platform CAAE 02091212.0.0000.5505. All patients underwent bilateral ACL reconstruction using semitendinosus and gracilis (STG) grafts. The graft from the STG was always harvested on the right side and soaked in saline, while on the left side, the graft was harvested and PRP (Magellan/Meditronic) was applied under fluoroscopy (Fig. 1). Patients responded to a postoperative pain questionnaire [Visual Analog Scale (VAS)] at 15, 30, and 60 days postoperatively. Magnetic resonance imaging (Siemens 1.5 T) was performed after 3 months, and isokinetic testing (Biodex) was conducted before and after 5 months of surgery to assess healing and injury functionality.

All patients underwent a physiotherapy protocol (analgesia and range of motion exercises within pain limits). Radiologists and isokinetic evaluators were blinded to whether saline or PRP was used on each side.

## Statistical analysis

Wilcoxon and Friedman tests were conducted for postoperative pain analysis, ANOVA for isokinetic peak torque values, and paired t-tests for muscle circumference values analysis.

## Results

In the VAS of pain, a subtle difference was observed with less pain on the left side at 15 and 30 days, but no difference at 60 days. However, statistically significant differences in pain intensity between the right and left sides were not found at 15 days (*P* = 0.4290), 30 days (*P* = 0.0578), and 60 days (*P* = 0.5637) (Table 1).

**Table 1:** Patient assessments of pain intensity measured by the VAS by side

**Side** (*n* = 12)	**Assessment**		
	**15 days**	**30 days**	**60 days**
**Right**			
Mean (sd)	6.5 (1.2)	3.6 (1.4)	0.5 (0.5)
Median	6.5	3.5	0.5
Minimum – Maximum	5–8	2–6	0–1
**Left**			
Mean (sd)	6.1 (1.1)	2.9 (1.2)	0.4 (0.5)
Median	6.0	3.0	0.0
Minimum - Maximum	5–8	1–5	0–1
**Right side** × **left side**	15d 30d	*P* = 0.4290 *P* = 0.0578	
(Wilcoxon test)	60d	*P* = 0.5637	
**Side**	15d × 30d × 60d	*P* = 0.0003	
**Right**			
(FriedmanTest)	15d × 30d	*P* = 0.0108	
	15d × 60d	*P* = 0.0108	
	30d × 60d	*P* = 0.0108	
**Side**	15d × 30d × 60d	*P* = 0.000	
**Left**			
(FriedmanTest)	15d × 30d	*P* = 0.0103	
	15d × 60d	*P* = 0.0106	
	30d × 60d	*P* = 0.0111	

Regarding the healing progression, statistically significant variations were found on both the right and left sides throughout the assessments (*P* = 0.0003 on both sides). On both sides, the reduction in pain intensity was significant on Day 30 compared to Day 15 (*P* = 0.0108 on the right and *P* = 0.0103 on the left) and on Day 60 compared to Day 30 (*P* = 0.0108 on the right and *P* = 0.0111 on the left).

In the isokinetic evaluation, there was no statistically significant difference in the preoperative Peak Torque (Table 2) and at 5 months postoperatively (Table 3) between the right and left sides (*P* = 0.8063), indicating similar behavior of Flexor Peak Torque over time (Table 4).

**Table 2:** Preoperative isokinetic assessment

	Peak torque flexor 60°/s	Peak torque flexor 60°/s
Name	Right	Left
ACSG	68	65
VJ	110	117
MACL	121	131
NO	69	83
RS	98	99
HAC	83	71
JP	105	110
AC	51	24
AB	100	102
MB	115	122
CV	73	85
GB	70	67

**Table 3:** Postoperative isokinetic assessment at 5 months

	Peak torque flexor 60°/s	Peak torque flexor 60°/s
Name	Right	Left
BAY	40	30
ACSG	51	24
VJ	64	79
MACL	130	156
NO	94	88
RS	90	95
HAC	75	70
JP	95	100
AB	105	107
MB	120	124
CV	75	88
GB	75	72

**Table 4:** Patient assessments of peak torque flexor at 60°/s by side

**Side** (*n* = 8)	**Assessment**		
	**Preoperative**	**Postoperative 5 months**	**Post-pre variation**
**Right**			
Mean (sd)	87.4 (25.6)	79.9 (28.6)	−7.5 (20.4)
Median	90.5	82.5	−8.0
Minimum - Maximum	45–121	40–130	−46 – 25
**Left**			
Mean (sd)	89.0 (31.2)	80.3 (41.8)	−8.8 (21.8)
Median	91.0	83.5	−5.0
Minimum - Maximum	36–131	24–156	−41–25
ANOVA			
**Side effect**		*P* = 0.8075	
**Time effect**		*P* = 0.2865	

There was no statistically significant effect of the Side factor (*P* = 0.8075), indicating similar averages of Peak Torque on both sides. No statistically significant effect of the Time factor was found (*P* = 0.2865), indicating similar averages of Peak Torque in the evaluations (Table 4).

As for the measured area in the posterior thigh musculature post-surgery, no statistically significant differences were found between the right and left sides in total muscle area (*P* = 0.6895), gracilis (*P* = 0.9083), semimembranosus (*P* = 0.7549), semitendinosus (*P* = 0.1733), and biceps (*P* = 0.2671) (Table 5).

**Table 5:** Patient assessments of muscle areas by side

**Muscle area** (*n* = 8)	**Side**	
	**Right**	**Left**
**Total**		
Mean (sd)	140.5 (7.3)	139.6 (8.4)
Median	138.6	138.9
Minimum - Maximum	132.1–155.0	128.0–152.0
*P*-value (paired t-test)	0.6895	
**Gracilis**		
Mean (sd)	5.3 (1.4)	5.3 (1.3)
Median	5.8	4.9
Minimum - Maximum	3.5 – 6.8	3.5 – 7.2
*P*-value (paired t-test)	0.9083	
**Semimembranosus**		
Mean (sd)	11.8 (1.8)	11.6 (2.3)
Median	11.8	12.2
Minimum - Maximum	8.0 – 13.6	7.4 – 14.0
*P*-value (paired t-test)	0.7549	
**Muscle area** (*n* = 8)	**Side**	
	**Right**	**Left**
**Semitendinosus**		
Mean(dp)	7.9 (2.3)	7.2 (1.9)
Median	7.1	6.7
Minimum - Maximum	5.5 – 11.0	4.9 – 10.0
*P*-value (paired t-test)	0.1733	
**Biceps**		
Mean (sd)	15.4 (2.2)	16.0 (2.4)
Median	16.1	16.2
Minimum - Maximum	12.0 – 18.1	11.2 – 19.2
*P*-value (paired t-test)	0.2671	

## Discussion

In contrast to studying muscle injuries in animals, many isogenic (same genetic background) and similarly induced lesions, the study of muscle injuries in humans faces the primary challenge of dealing with heterogeneous injuries. This heterogeneity arises due to the difficulty in reproducing or comparing injuries, given their variations in size and location.

The most common injuries, especially in our field, are injuries to the posterior thigh muscles related to sports such as soccer, athletics, rugby, basketball, handball, etc. The frequency of this injury in a season can be up to 30% compared to 0.4 per season in the case of ACL injuries. Therefore, muscle injuries in these sports require better understanding regarding the origin of the injury and treatment [13].

The idea of studying patients who have undergone bilateral ACL reconstruction surgery arises from the fact that it represents similar muscle injuries performed in the same manner and on the same individual. After the removal of the semitendinosus graft, the healing of the local stump will begin, which can be monitored clinically, through imaging (MRI), and with an isokinetic dynamometer. In general, studies involving muscle injury healing in humans follow injuries of varying patterns, sizes, and locations [13].

Other studies have previously addressed the use of PRP in muscle injuries in humans. Wright-Carpenter’s work [16] showed a better return to sport in athletes after the use of PRP, but there was no control or comparative group with MRI or imaging methods. An isokinetic dynamometer was also not used.

The study by Hamid *et al*. [17] evaluated the effects of autologous PRP on the time to return to sports activities. Randomly selected patients with an average age of 18 years and grade II posterior thigh muscle injuries were studied. The conclusion was that there was an improvement in the time to return to sports activity, allowing for recommendations of this treatment option for grade II tendon injuries.

Analyzing the retrospective study by Wetzel *et al*. [18], which involved 17 posterior thigh muscle injuries, 12 injuries failed conservative treatment and were subsequently treated with PRP applied at the muscle origin, while the other 5 injuries were treated conservatively only. In the PRP-treated group, there was a reduction in the VAS and the Nirschl Phase Rating Scale, but both groups returned to the same level of activity without major complications.

Reurink *et al*. [19], published in 2014, as well as the study by Moca MJ published in 2015, both randomized controlled trials, showed no difference in the use of PRP regarding return to sports and muscle injury recovery.

Bubnov *et al*. [20] showed improvement in pain and range of motion up to the 28th day; after the 28th day, the results did not demonstrate significant importance in the use of PRP.

There are numerous studies showing the influence of PRP on muscle injuries *in vitro* or in animals. The work by Hamid *et al*. [17], a systematic review published in 2014, demonstrated accelerated healing in animals; however, evidence in human studies is still limited.

Like Yoshitsugu Takeda *et al*. [21], whose study shows the regeneration of the tendons of the goose foot (gracilis and semitendinosus) below the joint line, after they were removed for ACL reconstruction.

However, if PRP can help, what is the optimal time for application, the appropriate quantity, or the real function of the hematoma, for example? [18]. These questions remain unanswered and we need future studies for clarification.

Other factors that can also influence muscle healing and are present in PRP have been described recently: cytokines such as interleukins and tumor necrosis factor (TNF) [22].

Factors that could negatively influence the study of bilateral ACL reconstruction include the possibility of a systemic effect of PRP, in addition to the local effect, which could influence the other side of the thigh. This is demonstrated in the work by Schippinger *et al*. [23], where the systemic effect of intramuscularly applied PRP was evaluated by collecting blood samples. No significant increase in growth factors was found, except for TGF-beta2. However, little is known about the systemic influence of intramuscularly applied PRP in terms of distant effects.

The literature contains various studies demonstrating the theoretical benefits of PRP use in muscle injuries [22, 24, 25]; however, these do not present statistically significant results that support its use in clinical practice.

The limiting factor of this case series is the number of patients studied (12 patients / 24 infiltrations), given that bilateral ACL injury is not common. Additionally, the potential systemic effects of PRP are unknown, which could interfere with the results on the side considered as the control.

We believe that a very important factor was performing the treatment with PRP and saline on very similar muscle injuries using the same instrument. One of the main problems in studying muscle injuries is the heterogeneity of the injuries, which complicates comparison and the results obtained.

## Conclusion

There was a slight difference (not statistically significant) at 15 and 30 days on the side where PRP was applied. No difference was observed in muscle circumference and strength at 3 and 5 months, respectively. Therefore, the decision was made to continue the study, increasing the number of cases, with the aim of publishing the obtained results.
